# NOX5: Molecular biology and pathophysiology

**DOI:** 10.1113/EP086204

**Published:** 2019-03-18

**Authors:** Rhian M. Touyz, Aikaterini Anagnostopoulou, Francisco Rios, Augusto C. Montezano, Livia L. Camargo

**Affiliations:** ^1^ Institute of Cardiovascular and Medical Sciences BHF Glasgow Cardiovascular Centre University of Glasgow Glasgow UK

**Keywords:** cancer, cardiovascular disease, kidney disease, Nox isoforms, oxidative stress, reactive oxygen species

## Abstract

**New Findings:**

- **What is the topic of this review?**
  This review provides a comprehensive overview of Nox5 from basic biology to human disease and highlights unique features of this Nox isoform
- **What advances does it highlight?**
  Major advances in Nox5 biology relate to crystallization of the molecule and new insights into the pathophysiological role of Nox5. Recent discoveries have unravelled the crystal structure of Nox5, the first Nox isoform to be crystalized. This provides new opportunities to develop drugs or small molecules targeted to Nox5 in an isoform‐specific manner, possibly for therapeutic use. Moreover genome wide association studies (GWAS) identified Nox5 as a new blood pressure‐associated gene and studies in mice expressing human Nox5 in a cell‐specific manner have provided new information about the (patho) physiological role of Nox5 in the cardiovascular system and kidneys. Nox5 seems to be important in the regulation of vascular contraction and kidney function. In cardiovascular disease and diabetic nephropathy, Nox5 activity is increased and this is associated with increased production of reactive oxygen species and oxidative stress implicated in tissue damage.

**Abstract:**

Nicotinamide adenine dinucleotide phosphate (NADPH) oxidases (Nox), comprise seven family members (Nox1–Nox5 and dual oxidase 1 and 2) and are major producers of reactive oxygen species in mammalian cells. Reactive oxygen species are crucially involved in cell signalling and function. All Noxs share structural homology comprising six transmembrane domains with two haem‐binding regions and an NADPH‐binding region on the intracellular C‐terminus, whereas their regulatory systems, mechanisms of activation and tissue distribution differ. This explains the diverse function of Noxs. Of the Noxs, NOX5 is unique in that rodents lack the gene, it is regulated by Ca^2+^, it does not require NADPH oxidase subunits for its activation, and it is not glycosylated. NOX5 localizes in the perinuclear and endoplasmic reticulum regions of cells and traffics to the cell membrane upon activation. It is tightly regulated through numerous post‐translational modifications and is activated by vasoactive agents, growth factors and pro‐inflammatory cytokines. The exact pathophysiological significance of NOX5 remains unclear, but it seems to be important in the physiological regulation of sperm motility, vascular contraction and lymphocyte differentiation, and NOX5 hyperactivation has been implicated in cardiovascular disease, kidney injury and cancer. The field of NOX5 biology is still in its infancy, but with new insights into its biochemistry and cellular regulation, discovery of the NOX5 crystal structure and genome‐wide association studies implicating NOX5 in disease, the time is now ripe to advance NOX5 research. This review provides a comprehensive overview of our current understanding of NOX5, from basic biology to human disease, and highlights the unique characteristics of this enigmatic Nox isoform.

## 1 INTRODUCTION

Nicotinamide adenine dinucleotide phosphate (NADPH) oxidases (Nox) are a family of transmembrane proteins that transfer electrons across membranes. In an NADPH‐dependent manner, Noxs catalyse the reduction of O_2_ to produce superoxide (O_2_^−^) (NADPH + 2O_2_ → NADP^+^ + H^+^ + 2O_2_^−^), which in turn dismutates to generate hydrogen peroxide [H_2_O_2_; spontaneously or catalysed by superoxide dismutase (SOD); Bedard & Krause, 2007; Maghzal, Krause, Stocker, & Jaquet, 2012]. This cascade of reactions leads to generation of secondary reactive oxygen species (ROS), including the reaction of O_2_^−^ with nitric oxide (NO) to form peroxynitrite, the iron‐catalysed Fenton reaction to produce hydroxyl radical (OH^−^), and peroxidase‐catalysed generation of hypochlorous acid (HOCl; Maghzal et al., 2012).

**Figure 1:**
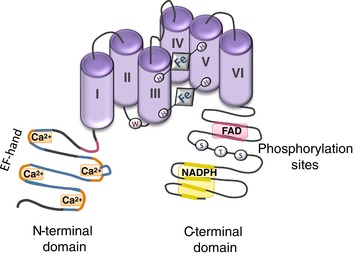
Diagram demonstrating structure of NOX5. NOX5 possesses six transmembrane domains with two haem‐binding sites, an N‐terminal domain with EF hands and a C‐terminal domain with phosphorylation sites (Bedard & Krause, 2012; Chen, Wang et al., 2015; Maghzal et al., 2012; Sedeek et al., 2012)

To date, seven mammalian Noxs have been identified, including Nox1–Nox5, dual oxidase 1 (duox1) and dual oxidase 2 (duox2) (Sedeek et al., 2012). All Noxs share structural homology in that they possess six transmembrane domains with two haem‐binding regions containing histidine residues and a NADPH‐binding region on the intracellular C‐terminus, which facilitates O_2_^−^ production (Leto, Morand, Hurt, & Ueyama, 2009; Figure 1). Noxs are differentially regulated, heterogeneously expressed and functionally distinct (Bedard, Lardy, & Krause, 2007; Lassègue, San Martín, & Griendling, 2012). Unlike Nox1–Nox4, duox1 and duox2, which have been well characterized, there is a paucity of information about NOX5, the most recently discovered Nox (Bánfi et al., 2001; Cheng, Cao, Xu, van Meir, & Lambeth, 2001). Here, a comprehensive review of the current knowledge of NOX5 is presented, with a focus on the unique characteristics of this unusual oxidase. To contextualise NOX5 within the Nox family, we provide a brief overview of Nox functions and Nox isoforms.

## 2 Noxs, REACTIVE OXYGEN SPECIES, PROTON PUMPS AND INTRACELLULAR pH

Noxs are enzymes that constitute a multifunctional and diverse group that are tightly regulated by many NADPH oxidase subunits and binding proteins in numerous cell types and tissues. During Nox activation, the electron donor NADPH provides two electrons that are translocated across the membrane to reduce O_2_ to O_2_^−^, hence the primary function of Noxs is the generation of O_2_^−^ (Bedard & Krause, 2007; Maghzal et al., 2012). However, in this process two protons are produced, which influence intracellular pH (pH_i_), and accordingly, it has been suggested that Noxs might have a dual function: firstly, the transport of electrons to generate ROS; and secondly, the conductance of protons across membranes (DeCoursey, Cherny, Zhou, & Thomas, 2000; Lamb, Moreland, & Miller, 2009; Vignais, 2002). Nox activation generates large quantities of ROS, especially in phagocytes, leading to depolarization and decreased pH_i_, which are direct effects of oxidase activity. In the 1980s, it was suggested that Nox2 itself is a phagocyte proton pump (Henderson, Chappell, & Jones, 1987); however, this notion could not be confirmed, and more recently, specific voltage‐gated proton channels (H_V_1), associated with Noxs, were found to be responsible for conductance of protons (DeCoursey, 2016). It seems that Nox moves electrons, whereas H_V_1 moves protons. These transporters, which are closely interrelated, have a symbiotic association, in which H_V_1 is required for optimal generation of ROS by Nox2, and H_V_1 is influenced by Nox‐induced electrogenic H^+^ efflux (DeCoursey, 2016; Seredenina, Demaurex, & Krause, 2015). Although most studies showing this association have focused on Nox2, other Noxs, and especially NOX5, might also be regulated by H_V_1 and pH (Seredenina et al., 2015).

## 3 A PRIMER ON Nox ISOFORMS

Nox2, also called gp91phox‐containing Nox and typically expressed in phagocytic cells, is the prototype NADPH oxidase and was the first to be identified (Gabig & Babior, 1979). In its activated state, Nox2 associates with the transmembrane protein p22phox, three cytosolic subunits (p47phox, p67phox and p40phox) and the small G proteins Rac1 or Rac2 (Dang, Cross, & Babior, 2001). The molecular weight of Nox2 is 58 kDa, but because it is highly glycosylated it appears as a smear of bigger size, ∼91 kDa on western blot (Paclet, Henderson, Campion, Morel, & Dagher, 2004). It should be highlighted that ‘Nox’ specifically refers to the electron‐transporting element of the enzyme (gp91phox) but is commonly used to denote the complete multi‐unit oxidase (DeCoursey, 2016). Nox2 is typically found in neutrophils, monocytes, macrophages and other phagocytic cells, but is also expressed in cells of the lung, heart, skeletal muscle and vasculature (Ferreira & Laitano, 2016; Sirker et al., 2016; Touyz et al., 2002). In phagocytic cells, Nox2 generates bursts of O_2_^−^, important in host defence responses (Touyz et al., 2002). Its essential clinical function is observed in patients with chronic granulomatous disease, an immunodeficiency syndrome caused by defective phagocytic Nox (Casimir et al., 1992). The other Nox isoforms have a heterogeneous distribution in multiple cell types.

Nox1, the first Nox2 homologue to be discovered, requires cytosolic subunits p47phox (or the homologues NOXO1β and NOXO1γ) and p67phox (NOXA1) for its activation (Suh et al., 1999; Takeya et al., 2003). It is most abundant in colon, prostate, uterus and vascular cells, and its expression is markedly increased in cancer cells (Juhasz et al., 2017; Parascandolo & Laukkanen, 2019). Nox1 was originally called Mox1 for ‘mitogenic oxidase’ because of its role in cell proliferation and mitogenesis (Suh et al., 1999). Nox1 is also an important driver of inflammation and fibrosis, and Nox1/4 inhibitors (GKT136901 and GKT137831) delay disease progression in experimental models of chronic inflammatory and fibrotic diseases (Teixeira et al., 2017). GKT137831 is currently being tested clinically in patients with primary biliary cholangitis, pulmonary fibrosis and liver fibrosis.

Nox3 is typically found in the inner ear and regulates vestibular function in an ROS‐dependent manner (Rousset, Carnesecchi, Senn, & Krause, 2015). Nox3 is also found in low abundance in the brain, lung and in fetal tissue, but the function in these tissues is unclear (Michihara, Oda, & Mido, 2016).

Nox4 is highly expressed in the kidney and in osteoclasts, fibroblasts, cardiomyocytes, endothelial and vascular smooth muscle cells (Yang et al., 2018; Zhang et al., 2018). It is also abundant in tumour cells and has been considered an oncoprotein (Graham et al., 2010). Similar to Nox1, Nox2 and Nox3, Nox4 requires p22phox for its activation and maturation, as demonstrated in human embryonic kidney 293 (HEK293) cells, in which Nox4 was inactive when p22phox was knocked out by CRISPR/Cas9 (Prior et al., 2016). Nox4 does not require p47phox and p67phox cytosolic subunits for its activation, but is regulated by polymerase δ‐interacting protein 2 (Poldip2), a multifunctional protein (Hernandes, Lassègue, & Griendling, 2017; Vukelic et al., 2018) and tyrosine kinase substrate 5 (Tks5) (Diaz et al., 2009). Unlike other Noxs, Nox4 produces both O_2_^−^ and H_2_O_2_ and is constitutively active. The H_2_O_2_‐generating potential is attributed to a histidine residue within an extracytosolic loop, rendering Nox4 with dehydrogenase function, which promotes electron transfer from NADPH to FAD and consequent H_2_O_2_ formation (Takac et al., 2011). Nox4 is associated with focal adhesions (Lyle et al., 2009), is important in cell migration and has been demonstrated in the nucleus, mitochondria and endoplasmic reticulum (ER), where its function remains unclear, although the ER might be a site of synthesis or post‐translational modification (Laurindo, Araujo, & Abrahão, 2014; Santos et al., 2014). In the endothelium, Nox4‐derived H_2_O_2_ has been considered to be an endothelium‐derived relaxing factor, causing vasodilatation (Liu, Bubolz, Mendoza, Zhang, & Gutterman, 2011). Studies in *Nox4* knockout and Nox4 overexpressing mice have shown that Nox4 is both cardiovascular protective and injurious (Morawietz, 2018; Schürmann et al., 2015). Suggestions have also been made that NOX4 dimerizes with NOX5, but mechanisms remain unclear (Kawahara, Jackson, Smith, Simpson, & Lambeth, 2011; Montezano et al., 2011).

Duox1 and Duox2 are Ca^2+^‐activated Noxs that localize primarily in epithelial cells at mucosal surfaces and are highly expressed in the thyroid gland. Duox1/2 play an important role in thyroid hormone biosynthesis (Carvalho & Dupuy, 2017) and have recently been shown to be involved in mediating innate immune responses (van der Vliet, Danyal, & Heppner, 2018).

NOX5, which has a more widespread distribution with unique characteristics, is the focus of the present review and is discussed in detail below.

## 4 NOX5, AN UNUSUAL MEMBER OF THE Nox FAMILY

NOX5 was discovered in 2001 by the Lambeth (Cheng et al., 2001) and Krause (Bánfi et al., 2001) laboratories and is the most recently characterized member of the Nox family. On western blot it is an 85 kDa protein, which is consistent with its predicted molecular mass (Bánfi et al., 2001; Cheng et al., 2001). Similar to other Noxs, NOX5 has six conserved predicted transmembrane α‐helices containing putative haem‐binding regions and a flavoprotein homology domain containing predicted binding sites for FAD and NADPH on the intracellular C‐terminus (Bánfi et al., 2001; Biberstine‐Kinkade et al., 2001; Cheng et al., 2001). However, it has many unusual features that distinguish it from the other Nox family members (Bedard, Jaquet, & Krause, 2012). Accordingly, we define NOX5 as the ‘enigmatic Nox’ (Table 1). Characteristics that distinguish NOX5 include the following: (i) the *NOX5* gene is absent in rodents, yet it is present in lower forms and mammals; (ii) it generates O_2_^−^ from a single gene product; (iii) it does not require any NADPH oxidase subunits for its activation; (iv) it has a unique N‐terminal extension that contains three or four Ca^2+^‐binding helix–loop–helix structure domains (EF hand); (v) NOX5 activation is highly sensitive to changes in intracellular free Ca^2+^ concentration ([Ca^2+^]_i_), as shown in cell studies where NOX5 is unable to generate O_2_^−^ in Ca^2+^‐free conditions; (vi) NOX5 is regulated by post‐translational modifications, including phosphorylation and oxidation, but unlike Nox2 does not seem to be glycosylated and has been described as a ‘bona fide non‐glycoprotein’; and (vii) to date, NOX5 is the first and only NADPH oxidase to be crystallized (Magnani et al., 2017) and thus provides opportunities to design specific NOX5 inhibitors and activators, crucial for biomedical research and potentially for therapeutic utility.

**Table 1:** Unusual characteristics of NOX5

(i)	Gene is absent in rodents
(ii)	Activated by increased [Ca^2+^]_i_
(iii)	Unlike other Noxs, it is not *N*‐glycosylated
(iv)	Does not require p22phox for its activation
(v)	Activation is independent of NADPH oxidase subunits
(vi)	Activation involves conformational changes
(vii)	NOX5 is the only Nox isoform to be crystallized

## 5 EXPRESSION OF NOX5 IN PHYSIOLOGICAL AND PATHOLOGICAL CONDITIONS

The early NOX5 studies demonstrated that mRNA of *NOX5* is expressed in pachytene spermatocytes of the testis and in B‐ and T‐lymphocyte‐rich areas of the lymph nodes and spleen (Bánfi et al., 2001; Cheng et al., 2001). Whether NOX5 is expressed and functionally active in circulating phagocytic cells has been debated, because original studies failed to identify NOX5 in peripheral lymphocytes, whereas recent studies indicate that NOX5 is expressed in human monocytes and macrophages (Manea et al., 2015; Marzaioli et al., 2017) and that NOX5 regulates human monocyte differentiation into dendritic cells (Marzaioli et al., 2017). However, most of those studies were performed in monocyte/macrophage leukaemia cell lines (Marzaioli et al., 2017), and the physiological role of neutrophil NOX5 is unclear. In the developing zebrafish, Nox5 is expressed throughout the CNS and, together with Nox2, it might be important in neurodevelopment and regeneration (Weaver et al., 2018; Weaver, Leung, & Suter, 2016). This is confirmed in human oligodendrocytes, which require NOX5 for differentiation and maturation (Accetta et al., 2016).

NOX5 has also been demonstrated in many non‐immune cells and tissues, including placenta, bone marrow, uterus, stomach, skeletal muscle, cancer cells and hepatocytes and in cells of the cardiovascular system, such as cardiomyocytes, endothelial and vascular smooth muscle cells (Mahbouli et al., 2017; Montezano et al., 2010; Yeung et al., 2016). The functional significance of this widespread expression profile suggests that NOX5 is likely to be important in many (patho)physiological processes in multiple systems that involve ROS. In particular, in physiological conditions, NOX5 has been implicated in the regulation of spermatozoa through redox‐dependent processes that control sperm motility, sperm–oocyte fusion, cell proliferation and cytokine secretion (Ghanbari, Keshtgar, & Kazeroni, 2018). Inhibition of NOX5 activity reduces human sperm mobility and viability (Ghanbari et al., 2018). NOX5 might also be important in lymphocyte function, and we identified a physiological role for NOX5 in vascular contraction (Montezano et al., 2018).

Cell migration, contraction and proliferation are Ca^2+^‐dependent processes, and this is particularly pertinent to NOX5, because NOX5 itself is regulated by Ca^2+^. The relationship between Ca^2+^, ROS and NOX5 is highlighted in NOX5‐expressing cells that generate large amounts of O_2_^−^ in response to increasing concentrations of intracellular Ca^2+^ (Banfi et al., 2001). In these conditions, NOX5 might also act as a proton channel, possibly to balance changes in charge and pH secondary to electron export involved in O_2_^−^ production, as discussed in detail above.

## 6 REGULATION OF NOX5

**Figure 2:**
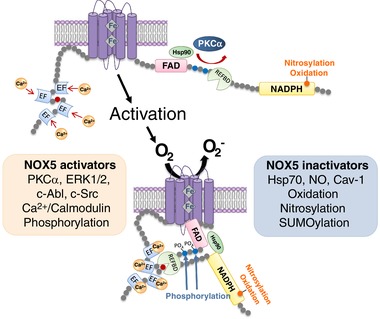
Schematic diagram demonstrating regulatory mechanisms of NOX5. NOX5 is activated when the [Ca^2+^]_i_ increases and in response to various activators, including regulatory proteins (calmodulin), kinases (PKCα, ERK1/2, CAM kinase II and c‐Abl) and through post‐translational modifications (phosphorylation). NOX5 is inactivated by regulatory proteins [caveolin‐1 (Cav‐1)] and chaperone molecules [heat shock protein 70 (Hsp70)] and through post‐translational modifications (oxidation, nitrosylation and SUMOylation) (Chen, Haigh et al., 2015; Chen, Wang et al., 2015; Chen, Yin, Dimitropoulou, & Fulton, 2016; Fulton, 2009)

NOX5 plays a major role in O_2_^−^ generation in various cell types, hence NOX5 activation needs to be regulated tightly to maintain cellular redox status. NOX5 activation involves numerous regulatory processes, including genetic factors, changes in [Ca^2+^]_i_, phosphorylation and interaction with regulatory proteins. On the contrary, inactivation of NOX5 seems to involve post‐translational modifications, especially oxidation, *S*‐nitrosylation and SUMOylation (Figure 2).

### 6.1 Regulation of *NOX5* gene

In humans, the *NOX5* gene is located on chromosome 15, with six isoforms having been identified [NOX5α, β, γ, δ, ε (also called short NOX5, NOX5S) and ζ; Serrander et al., 2007]. NOX5α, NOX5β and NOX5γ are functionally active and generate ROS. NOX5α and NOX5β are the major isoforms expressed in human cells and seem to be negatively regulated by NOX5ε, which inhibits NOX5‐induced ROS production (Fulton, 2009). NOX5δ, NOX5ε and Noxζ do not seem to produce appreciable amounts of O_2_^−^, and their functional significance is unclear. Epigenetic factors, including overexpression of histone deacetylase 2, cause upregulation of the *NOX5* gene promotor activity in vascular smooth muscle cells (Manea, Todirita, Raicu, & Manea, 2014).

Within the coding sequence of the human *NOX5* gene, a number of polymorphisms have been described. To assess whether these impact enzymatic activity, Wang, Chen, Le, Stepp, & Fulton (2014) investigated how single nucleotide polymorphisms within the coding region of *NOX5* influence ROS production by studying Cos cells expressing various *NOX5αβ* mutants. They demonstrated that exonic single nucleotide polymorphisms in *NOX5* influence oxidase activity by reducing, rather than by increasing, enzymatic function (Wang et al., 2014).

### 6.2 NOX5 regulatory proteins

Although NOX5 does not require NADPH oxidase subunits for its activation, it is influenced by various regulatory proteins, some of which interact directly with NOX5, including protein kinase C (PKC), calmodulin, caveolin‐1, c‐Abl1 and chaperone molecules (Hsp90 and Hsp70) (Chen, Yu, et al., 2014; Chen et al., 2015). These interactions influence NOX5 activity differentially and might also stabilize the enzyme. For example, PKC and calmodulin increase NOX5 sensitivity to Ca^2+^, promoting activation, whereas interaction with caveolin‐1 is associated with NOX5 inactivation (Chen, Yu, et al., 2014; Chen et al., 2015; El Jamali et al., 2008). Pro‐inflammatory transcription factors, such as nuclear factor‐κB, AP‐1 and STAT1/STAT3, have also been shown to regulate NOX5 in human aortic smooth muscle cells (Pandey & Fulton, 2011).

### 6.3 NOX5 activation is Ca^2+^ dependent

One of the distinguishing features about NOX5 is its dependence on Ca^2+^ for its regulation (Bánfi et al., 2001). Activation of NOX5 in response to elevated Ca^2+^ is a multi‐phased process (Tirone, Radu, Craescu, & Cox, 2010). The first phase involves an increase in [Ca^2+^]_i_, followed by Ca^2+^ binding to the EF hand on the N‐terminal region. This causes conformational changes that lead to exposure of hydrophobic areas. The regulatory domain then binds to the catalytic domain in the C‐terminus, causing its activation. The amount of Ca^2+^ necessary to activate NOX5 fully is high, and accordingly, additional systems involving regulatory proteins are operational that increase sensitivity to Ca^2+^, thereby facilitating ROS generation at lower [Ca^2+^]_i_. Hence, NOX5 can be activated directly by Ca^2+^ or indirectly by interacting with other proteins and kinases, such as Ca^2+^‐bound calmodulin or PKC (Wei et al., 2012).

### 6.4 Post‐translational modification of NOX5

Post‐translational modification of Noxs is not Nox specific, because Nox1, Nox2, Nox4 and NOX5 have been found to be phosphorylated, oxidized and nitrosylated. Nox1–Nox4, but not NOX5, also undergo glutathionylation.

#### 6.4.1 NOX5 phosphorylation

The C‐terminal domain of NOX5 contains a group of serine and threonine residues (Ser475, Ser490, Ser494, Ser 498, Ser516 and T5120) that are phosphorylated in response to various kinases, including PKCα (Jagnandan et al., 2007), ERK1/2, c‐Src, Abl1 and Ca^2+^–calmodulin‐dependent protein kinase II (CAM kinase II) (Jha, Watson, Mathew, de Vos, & Jandeleit‐Dahm, 2017). Recent studies in podocytes showed that NOX5 is a downstream target of the Toll‐like receptor pathway and that NOX5‐induced ROS generation is modulated by IRAK1/4 activity; processes that are likely to involve phosphorylation of the oxidase (Holterman et al., 2018). NOX5 phosphorylation increases sensitivity to Ca^2+^, which increases oxidase activity.

#### 6.4.2 NOX5 oxidation

Recent studies suggest that oxidation of NOX5 is associated with inactivation of the enzyme. NOX5 contains cysteine and methionine residues, which are highly sensitive to oxidation (Pendyala & Natarajan, 2010). Using isothermal titration calorimetric methods, Petrushanko et al. (2016) demonstrated that in the presence of increased ROS, cysteine and methionine residues in the Ca^2+^‐binding (EF) domain of NOX5 are oxidized, which causes a decrease in bound Ca^2+^. They also showed by ultraviolet circular dichroism spectroscopy that oxidation decreases NOX5 α‐helical content and alters the secondary and tertiary structure of NOX5 (Petrushanko et al., 2016). These processes lead to a decrease in stoichiometry of the binding domain for Ca^2+^, with a consequent decrease in enzymatic activation. This phenomenon might act as a potential cellular protective mechanism against excessive NOX5‐induced ROS generation and oxidative stress.

#### 6.4.3 NOX5 nitrosylation

Using the biotin switch assay and mass spectrometry, NOX5 was found to be N‐nitrosylated on four major sites: C107, C246, C519 and C694 (Qian et al., 2012). When exposed to NO, NOX5 is nitrosylated, resulting in reduced oxidase activity and decreased O_2_^−^ production. This is a reversible process. Exogenous and endogenously generated NO decrease NOX5‐induced ROS production in a dose‐dependent manner; effects that are blocked by NO synthase inhibitors (Qian et al., 2012). This NO synthase–NO NOX5 effect is dependent on nitrosylation, but not on phosphorylation or glutathiolation. Hence, NO might protect against excessive NOX5‐induced ROS generation and contribute to redox balance, especially in conditions associated with oxidative stress. Nitrosylation of NOX5 has also been demonstrated in insect models (Oliveira, Lieberman, & Barillas‐Mury, 2012).

#### 6.4.4 SUMOylation of NOX5

SUMOylation is a form of post‐translational modification of proteins that functions as a molecular competitor of ubiquitination. It involves a member of the small ubiquitin‐like modifier (SUMO) family of proteins that conjugates to lysine in target proteins (Yang et al., 2010). Increased expression of SUMO1 is associated with decreased NOX5 activity, whereas inhibition of SUMO1 caused an increase in NOX5‐induced O_2_^−^ production (Pandey et al., 2011). The mechanisms underlying these processes are unclear, but NOX5 does not seem to be a direct target for post‐translational SUMOylation.

#### 6.4.5 Palmitoylation of NOX5

NOX5 might also be palmitoylated (Touyz RM, Fuller W; unpublished data), although direct evidence for this is still awaited. Protein *S*‐palmitoylation is a reversible post‐translational modification that influences subcellular localization, trafficking and function of proteins (Oddi et al., 2017).

### 6.5 Intracellular trafficking of NOX5

Nox1, Nox2, Nox3 and Nox4 are primarily associated with the cell membrane, in large part because of their obligatory need for cell membrane‐associated p22phox (Bedard & Krause, 2007; Maghzal et al., 2012). In contrast, NOX5 is expressed mainly in intracellular compartments localized mainly in the perinuclear area and ER (Ahmarani et al., 2013). The reason why NOX5 is abundant in these areas is unclear, but the ER is a site of protein synthesis and post‐translational modification. Also, ER function is redox sensitive, and the ER is a rich store of intracellular Ca^2+^ important for NOX5 activation. Hence, the ER–NOX5 association might be important in NOX5 regulation, and the ER might be a region of cross‐talk between Ca^2+^‐ and redox‐sensitive signalling through NOX5 (Montezano et al., 2018).

**Figure 3:**
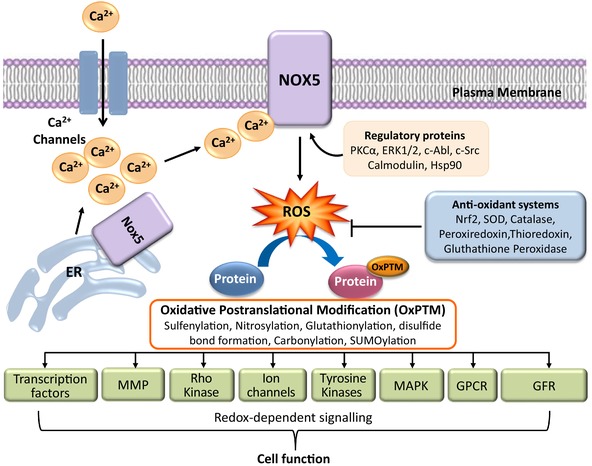
Diagram demonstrating the role of NOX5 in cell signalling and function. NOX5 is activated by Ca^2+^ and other regulatory mechanisms, and traffics from the perinuclear region and endoplasmic reticulum (ER) to the plasma membrane, where it catalyses the reduction of O_2_ to produce superoxide (O_2_^−^), which in turn dismutates to generate hydrogen peroxide [H_2_O_2_; spontaneously or catalysed by superoxide dismutase (SOD)]. Levels of pro‐oxidant reactive oxygen species (ROS) are normally controlled by numerous antioxidant systems, which maintain redox balance. Increased generation of ROS causes oxidative post‐translational modifications (OxPTM) of numerous molecules that influence redox signalling and cell function. Abbreviations: GFR, growth factor receptor; GPCR, G protein‐coupled receptor; MAPK, mitogen‐activated protein kinases; and MMP, matrix metalloproteinases

Within cells, NOX5 is dynamic and traffics from intracellular sites to the cell membrane, where it may associate with cholesterol‐rich microdomains (caveolae/lipid rafts), bringing it into close proximity to regulatory proteins, such as PKC, that influence its activation (Anagnostopoulou, Persson, Montezano, & Touyz, 2017; Chen et al., 2014; Figure 3). In addition, co‐localization of NOX5‐derived ROS and redox‐sensitive molecules facilitates efficient signalling in these microdomains. The molecular mechanisms controlling NOX5 trafficking are unclear, because unlike other Noxs, which traffic from the ER to the plasma membrane through pathways that involve *N*‐glycosylation and Sar1/Stx5 signalling, NOX5 is not glycosylated (Kiyohara et al., 2018). Nevertheless, NOX5 trafficking seems to involve Sar1 without being glycosylated. Other mechanisms of NOX5 trafficking involve polybasic domains in the N‐terminus of NOX5, which bind to phosphatidylinositol 4,5‐bisphosphate, a multifunctional regulatory lipid in the plasma membrane that influences the temporal and spatial specificity of intracellular signalling pathways and vesicular and subcellular trafficking (Kawahara & Lambeth, 2008).

## 7 PATHOPHYSIOLOGY OF NOX5

NOX5‐derived ROS have been implicated in a number of pathologies, especially cardiovascular disease, renal disease and cancer. Other pathologies, such as neurodegenerative diseases (Tarafdar & Pula, 2018), pulmonary arterial hypertension (Peng, Liu, Xu, Peng, & Luo, 2017) and liver fibrosis (Andueza et al., 2018), have also been linked to NOX5, but there is very little supporting evidence for this.

### 7.1 NOX5 and cardiovascular diseases

Calcium‐ and redox‐dependent signalling play a major role in cardiac and vascular contraction and function, hence Ca^2+^‐regulated NOX5‐induced ROS generation might be especially relevant in these systems. We recently defined NOX5 as a pro‐contractile Nox isoform that influences the molecular contractile machinery in vascular smooth muscle cells (Montezano et al., 2018). We also demonstrated that NOX5 regulates vascular contraction in NOX5‐expressing mice and that it is essential for smooth muscle contraction in arthropod models (Montezano et al., 2018), supporting earlier studies in *Drosophila* (Ritsick, Edens, Finnerty, & Lambeth, 2007). In the heart, NOX5 plays a role in the regulation of intermediate‐conductance Ca^2+^‐activated K^+^ channels (KCNN4), important for coronary artery smooth muscle cell contraction and progression of atherosclerosis (Gole, Tharp, & Bowles, 2014).

Among the first studies suggesting a role for NOX5 in the cardiovascular system was the demonstration that NOX5 protein is expressed in the ER and perinuclear area of human microvascular endothelial and vascular smooth muscle cells and that NOX5 is constitutively active, contributing to basal ROS production (Ahmarani et al., 2013; BelAiba et al., 2007). In humans, NOX5 is the primary vascular ROS‐generating Nox isoform and is stimulated by vasoactive agents (angiotensin II and endothelin‐1), growth factors (platelet‐derived growth factor and epidermal growth factor) and pro‐inflammatory mediators (transforming growth factor‐β and cytokines) (Jay et al., 2008; Manea, Manea, Florea, Luca, & Raicu, 2012; Montezano et al., 2010; Touyz, Anagnostopoulou, Camargo, Rios, & Montezano, 2019). Increased activation of endothelial cell NOX5 causes cell proliferation and formation of capillary‐like structures, important in atherosclerosis and angiogenesis (Guzik et al., 2008; Wang & Hartnett, 2017), and these processes are mediated via stromal cell‐derived factor‐1α and c‐Jun N‐terminal kinase 3 (Pi et al., 2014).

Many of the molecular and cellular processes involved in vascular remodelling associated with ageing, atherogenesis, hypertension and aneurysm formation, such as cell proliferation, inflammation and fibrosis, might involve NOX5‐dependent mechanisms (Guzik et al., 2013; Guzik, & Touyz, 2017). One of the best‐studied vasoactive regulators of NOX5 is angiotensin II, which increases expression and activation of the enzyme, in part through its effects on increasing [Ca^2+^]_i_ (Montezano et al., 2015). In contrast, activation of the vasoprotective axis of the renin–angiotensin system through angiotensin‐(1–7) prevents actin cytoskeleton reorganization and inhibits thrombin‐induced vascular inflammation by reducing expression and activity of NOX5 (Pai, Lo, Hsu, Peng, & Wang, 2017).

NOX5 has been demonstrated in human coronary artery disease, and its expression is increased in intramyocardial arteries in myocardial infarction (Hahn et al., 2012). In human atherosclerosis, oxidative and inflammatory processes involve increased expression and activation of NOX5 in vascular cells and resident macrophages (Chen et al., 2016; Hahn et al., 2012). In *in vitro* studies, exposure of monocytes and monocyte‐derived macrophages to increasing concentrations of interferon‐γ or oxidized low‐density lipoprotein, conditions that recapitulate atherogenesis, induced a dose‐dependent increase in NOX5 expression and ROS production (Manea et al., 2018). However, not all models of vascular inflammation and remodelling are associated with NOX5, as demonstrated in a primate model of atherosclerosis, where NOX2, but not NOX5, was involved in vascular injury (Stanic, Pandey, Fulton, & Miller, 2012).

Experimental models suggest that NOX5 also plays a role in the pathophysiology of stroke. In mice expressing human NOX5 in an endothelial cell‐specific manner, blood pressure was elevated and the risk of stroke increased (Kleikers et al., 2014). This seemed to be especially important in female mice, suggesting sexual dimorphism for NOX5‐related stroke. These studies suggested that targeting NOX5 might be vaso‐ and neuro‐rotective in conditions associated with elevated blood pressure and risk of stroke.

NOX5 has also been implicated in the development of hypertension. Kidney NOX5 expression and activity are increased in patients with essential hypertension, and NOX5 is likely to be a major cause of renal oxidative stress in hypertension (Holterman, Thibodeau, & Kennedy, 2015). We previously demonstrated increased blood pressure in mice expressing human NOX5 in the kidney (Holterman et al., 2014; Jha et al., 2017). However, the relationship between vascular NOX5 and blood pressure seems to be an age‐dependent phenomenon, because we found that in 16‐ to 20‐week‐old mice expressing human NOX5 specifically in vascular smooth muscle cells, blood pressure was not elevated (Montezano et al., 2018), whereas in 30‐ to 35‐week‐old mice, blood pressure was significantly increased (Montezano AC, Touyz RM; unpublished data). Clinically, a putative role for NOX5 in hypertension was recently unravelled in a genome‐wide association study searching for novel blood pressure‐associated genes (Kraja et al., 2017). In that study of 475,000 people, *NOX5* was identified as a putative blood pressure‐associated gene, especially linked to systolic blood pressure (Kraja et al., 2017). Other genetic studies have also shown associations between Nox and blood pressure. Findings from the Genetic Epidemiology Network of Salt‐Sensitivity study (Han et al., 2017) demonstrated that common variants of Nox‐related genes are associated with blood pressure responses to dietary sodium intervention in a Chinese population. However, more research in the field is needed to elucidate fully the role of NOX5 in the development of hypertension and associated cardiovascular disease.

### 7.2 NOX5 and kidney disease

NOX5 is expressed in numerous cell types in the kidney and seems to be the predominant Nox isoform in human renal proximal tubule cells (Chen et al., 2016). We were amongst the first to demonstrate that NOX5 is upregulated in human diabetic nephropathy and that it influences filtration barrier function and blood pressure through the generation of ROS (Holterman et al., 2014). These findings were supported by studies in mice expressing human NOX5 in a podocyte‐specific manner that exhibited podocyte dysfunction, albuminuria and hypertension, processes that were exacerbated when mice were made diabetic by treatment with streptozotocin (Jha et al., 2017). NOX5‐induced renal inflammation involves induction of cytokine expression and upregulation of Toll‐like receptors, which causes a feedforward system where Toll‐like receptor activation enhances NOX5‐induced generation of ROS and consequent oxidative stress and renal injury (Jha et al., 2017). More recently, we have shown that expression of human NOX5 in mice in a vascular smooth muscle/mesangial cell‐specific manner causes renal oxidative stress, glomerulosclerosis, mesangial expansion, renal inflammation and fibrosis, processes that accelerate progression of renal disease in diabetes (Jha et al., 2017). Other forms of renal disease are also associated with increased NOX5 expression, including sepsis‐induced acute kidney injury (Ge, Huang, Zhu, Bian, & Pan, 2017) and metabolic disease‐related renal damage (Wan, Su, & Zhang, 2016). Although NOX5 has been demonstrated in human kidney cells, the major renal Nox isoform is NOX4, which was originally called Renox (renal Nox). The relationship between NOX4 and NOX5 in the kidney is unclear, but NOX4 might regulate NOX5‐induced ROS production (Montezano et al., 2011).

### 7.3 NOX5 and cancer

Overexpression of Noxs and uncontrolled redox‐dependent cell proliferation have been demonstrated in various cancers (Gào & Schöttker, 2017; Roy et al., 2015). NOX5 expression and activity are increased in gastric cancer, malignant melanoma, breast cancer, prostate cancer and oesophageal cancer (Antony et al., 2017; Dho et al., 2017; Gào & Schöttker, 2017; Kalatskaya, 2016; Roy et al., 2015). Increased Nox activity and dysregulated production of ROS cause tissue injury, DNA damage and uncontrolled cell proliferation that are already evident in pre‐malignant conditions, especially Barrett's oesophagitis (Kalatskaya, 2016). Pathways implicated in these NOX5–ROS‐dependent processes include signalling molecules (MAP kinases, PI3K, PKC and p27Kip1), transcription factors (APE1/Ref‐1, hypoxia‐inducible factor‐1α, AP‐1, Nrf2, nuclear factor‐κB, p53, FOXO, STAT5A and β‐catenin) (Antony et al., 2017; Dho et al., 2017; Roy et al., 2015) and adaptor proteins (Ruk/CIN 85) (Bazalii, Horak, Pasi chn yk, Komisarenko, & Drobot, 2016).

### 7.4 NOX5 and cancer cell sensitivity to cisplatin

NOX5 has also been associated with sensitivity of cancer cells to chemotherapeutic drugs, such as cisplatin. In skin, breast and lung cancer cells, cisplatin treatment increased expression of NOX5, with an associated increase in ROS‐mediated cancer cell death (Dho et al., 2015); responses that seem to be dose dependent. Exposure of U937 histiocytic lymphoma cells to cisplatin also caused an increase in NOX5 expression (Park et al., 2018). When NOX5 was downregulated in these cells, sensitivity to cisplatin was increased through pathways that involve N‐Myc downstream‐regulated gene 2 (NDRG2) (Park et al., 2018). In human ovarian adenocarcinoma cells exposed to cisplatin, development of drug resistance was associated with increased gene expression of antioxidant enzymes (*SOD2*, *CAT*, *GPX1*, *HO‐1*) and the transcription factor Nrf2, and decreased expression of NOX5, suggesting an adaptive antioxidant response underlying molecular mechanisms associated with cancer cell resistance to cisplatin (Kalinina et al., 2018). Taken together, NOX5 has been suggested as a potential target of cancer cell sensitivity to chemotherapies, such as cisplatin.

## 8 CONCLUSIONS

All ROS‐generating Noxs are characterized by their ability to transport electrons across membranes and to produce O_2_^−^ and/or H_2_O_2_, which are important signalling molecules that influence all aspects of cell function (Chen, Wang, Barman, & Fulton, 2015). The regulation, mechanisms of activation and tissue distribution of the seven members of the Nox family are distinct. This is especially relevant for NOX5, which is unique in that it is absent in rodents, it generates ROS from a single gene product, it does not require any NADPH oxidase subunits for its activation, it has a unique N‐terminal extension that contains Ca^2+^‐binding domains, and it is not glycosylated. In physiological conditions, NOX5‐induced ROS generation seems to be important in the regulation of sperm motility, smooth muscle contraction and lymphocyte function, and in pathological conditions it has been implicated in cardiovascular disease, kidney disease and cancer. The field of NOX5 pathophysiology is still immature, but with advancements in NOX5 biochemistry and biology, the development of novel transgenic NOX5‐expressing experimental models, characterization of the NOX5 crystal structure and identification of *NOX5* mutations and polymorphisms, the significance of NOX5 in human health and disease will become more apparent.

## COMPETING INTERESTS

None declared.
